# Heterogeneous Integration of Freestanding Bilayer Oxide Membrane for Multiferroicity

**DOI:** 10.1002/advs.202207481

**Published:** 2023-04-03

**Authors:** Kyeong Tae Kang, Zachary J Corey, Jaejin Hwang, Yogesh Sharma, Binod Paudel, Pinku Roy, Liam Collins, Xueijing Wang, Joon Woo Lee, Yoon Seok Oh, Yeonhoo Kim, Jinkyoung Yoo, Jaekwang Lee, Han Htoon, Quanxi Jia, Aiping Chen

**Affiliations:** ^1^ Center for Integrated Nanotechnologies Los Alamos National Laboratory Los Alamos NM 87545 USA; ^2^ Department of Physics Kyungpook National University Daegu 41566 South Korea; ^3^ Department of Materials Design and Innovation University of Buffalo ‐ The State University of New York Buffalo NY 14260 USA; ^4^ Department of Physics Pusan National University Busan 46241 South Korea; ^5^ Center for Nanophase Materials Sciences Oak Ridge National Laboratory Oak Ridge TN 37831 USA; ^6^ Department of Physics Ulsan National Institute of Science and Technology Ulsan 44919 South Korea; ^7^ Interdisciplinary Materials Measurement Institute Korea Research Institute of Standards and Science (KRISS) Daejeon 34133 South Korea

**Keywords:** freestanding oxide membranes, magnetic anisotropy reorientation, magnetoelectric coupling, multiferroics

## Abstract

Transition metal oxides exhibit a plethora of electrical and magnetic properties described by their order parameters. In particular, ferroic orderings offer access to a rich spectrum of fundamental physics phenomena, in addition to a range of technological applications. The heterogeneous integration of ferroelectric and ferromagnetic materials is a fruitful way to design multiferroic oxides. The realization of freestanding heterogeneous membranes of multiferroic oxides is highly desirable. In this study, epitaxial BaTiO_3_/La_0.7_Sr_0.3_MnO_3_ freestanding bilayer membranes are fabricated using pulsed laser epitaxy. The membrane displays ferroelectricity and ferromagnetism above room temperature accompanying the finite magnetoelectric coupling constant. This study reveals that a freestanding heterostructure can be used to manipulate the structural and emergent properties of the membrane. In the absence of the strain caused by the substrate, the change in orbital occupancy of the magnetic layer leads to the reorientation of the magnetic easy‐axis, that is, perpendicular magnetic anisotropy. These results of designing multiferroic oxide membranes open new avenues to integrate such flexible membranes for electronic applications.

## Graphical abstract

Heterogeneous integration of oxides possessing multiferroicity is realized in a freestanding membrane. BaTiO_3_/La_0.7_Sr_0.3_MnO_3_ freestanding bilayer membrane is fabricated by using pulsed laser epitaxy. It exhibits room‐temperature ferroelectric ferromagnetism along with magnetoelectric coupling. Plus, owing to the absence of substrate strain, the ferromagnetism in La_0.7_Sr_0.3_MnO_3_ experiences the reorientation of the magnetic anisotropy.

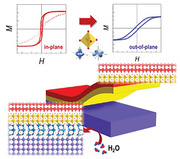

## 1 Introduction

The freestanding membrane structure is a new platform for the design and tuning of functionalities in transition metal oxides (TMOs).^[^ ^1^, ^2^, ^3^ ^]^ Oxide membranes with strongly correlated *d*‐electrons hold the potential for the expansion of quantum functionalities^[^ ^4^ ^]^ beyond 2D transition‐metal dichalcogenides with weakly interacting *s* and *p* orbitals. The fabrication of freestanding oxide membranes has been explored in some complex oxides.^[^ ^5^, ^6^ ^]^ This field has recently attracted considerable attention owing to the successful fabrication of freestanding TMO membranes using water‐soluble Sr_3_Al_2_O_6_ (SAO) as the sacrificial buffer layer.^[^ ^7^ ^]^ The SAO not only serves as a buffer layer for the epitaxial growth of some TMO films but also offers the feasibility to dissolve itself in water and fabricate freestanding TMO membranes. Therefore, high crystallinity TMO films or heterostructures can be formed into freestanding membranes. This method allows the exploration of novel techniques for designing the functionalities of TMOs. Quantum properties such as ferroelectricity and ferromagnetism have emerged in single‐layered oxide membranes.^[^ ^8^, ^9^, ^10^, ^11^, ^12^ ^]^ On one hand, the initial magnetic freestanding TMO membranes were synthesized by employing La_0.7_Sr_0.3_MnO_3_ (LSMO),^[^ ^7^ ^]^ La_0.7_Ca_0.3_MnO_3_,^[^ ^10^ ^]^ and SrRuO_3_.^[^ ^13^ ^]^ Owing to their excellent elasticity, the membranes can experience an enormous strain effect that modifies the quantum properties of oxides. For instance, ferromagnetism in the La_0.7_Ca_0.3_MnO_3_ membrane is manipulated by the application of uniaxial and biaxial strain.^[^ ^10^ ^]^ On the other hand, single‐crystalline BiFeO_3,_ a prototype multiferroic material, is realized in the freestanding membrane form to explore the effect of mechanical deformation on quantum properties.^[^ ^9^, ^14^, ^15^ ^]^ Astonishingly, the freestanding BiFeO_3_ membrane with two‐unit, which is even thinner than the critical thickness for ferroelectricity, exhibits ferroelectricity. Also, freestanding BaTiO_3_ (BTO) membrane preserving ferroelectricity was reported to realize ferroelectric tunneling junction devices.^[^ ^8^, ^16^ ^]^ Moreover, combining two or more oxide membranes gives rise to hybridized properties due to their interactions at the interface.^[^ ^17^, ^18^ ^]^ The observation of the topological Hall effect in the heterogeneous membranes of SrRuO_3_/SrIrO_3_ demonstrates the potential of utilizing the heterogeneous design in oxide membranes.^[^ ^19^ ^]^ Recently, studies have been focused on freestanding membranes of vertically aligned nanocomposite structures of oxides showing intriguing properties such as multiferroicity and exchange bias effects.^[^ ^20^, ^21^ ^]^

Among the various functionalities, multiferroics and magnetoelectric (ME) couplings have been widely studied because multiferroic materials are considered key materials for spintronic devices.^[^ ^22^ ^]^ Although magnetism requires partially filled *d*‐orbitals in transition metals, *d* ^0^‐ness is necessary for conventional ferroelectricity.^[^ ^23^, ^24^ ^]^ Single‐phase multiferroic materials with exotic mechanisms, for example, lone pair and geometric distortion, are rare.^[^ ^25^, ^26^, ^27^, ^28^ ^]^ Alternatively, ME coupling has often been achieved in heterogeneous multiferroics of multilayers and composites consisting of ferroelectric and ferromagnetic materials.^[^ ^29^ ^]^ The piezoresponse of ferroelectrics and the magnetostriction of ferromagnet can be tied when their corresponding layers are epitaxially coupled.^[^ ^30^, ^31^, ^32^ ^]^ A combination of BTO and LSMO is the most widely studied ME composite,^[^ ^33^, ^34^, ^35^ ^]^ as BTO shows robust ferroelectricity and LSMO exhibits ferromagnetism at a temperature higher than 300K. Furthermore, the sensitive nature of BTO to the external lattice modulation and the positive magnetostriction of LSMO could result in a strong ME coupling. To investigate the effect of strain on the multiferroicity in BTO/LSMO and BTO/CoFe_2_O_4_ thin films, a variety of substrates have been employed.^[^ ^36^, ^37^ ^]^

The substrate clamping effect is a major issue in ME multilayer thin films. Several methods have been proposed to address this challenge. For example, BTO‐CoFe_2_O_4_ vertically aligned nanocomposites with a reduced substrate clamping effect have been reported to show enhanced ME coupling. In addition, various flexible substrates, such as polymer‐type or Mica substrates, have been employed to fabricate flexible multiferroics.^[^ ^20^, ^38^ ^]^ However, these substrates make it difficult to grow high crystallinity multiferroic films due to the limitations of the substrate temperature and the crystal structure of the materials. As discussed earlier, the fabrication of flexible freestanding BTO and CoFe_2_O_4_ thin films has been reported.^[^ ^39^ ^]^ These studies pave new pathways for designing flexible multiferroic heterostructures for many potential applications. Although a multiferroic heterogeneous membrane of BTO/Fe has been reported to have good multiferroic properties,^[^ ^40^ ^]^ the strain effect in all‐oxide ME nanocomposites, made of freestanding heteroepitaxial oxide membranes, has not been studied thus far.

In this study, we fabricated a heterogeneous bilayer oxide membrane using the sacrificial buffer layer method. The bilayer membrane of BTO/LSMO shows simultaneous room‐temperature ferroelectricity and ferromagnetism. The freestanding membrane provides a distinct strain environment for both BTO and LSMO, leading to the reorientation of the magnetic anisotropy of the LSMO layer. Owing to the preserved heteroepitaxial coupling between the layers, the magnetostriction of LSMO favors a room‐temperature magnetoelectric behavior between the ferroelectric and ferromagnetic layers. Through density functional calculations, the strain change in LSMO can be used to reconstruct the orbital occupancy within the Mn atom. The resultant shift in magnetic anisotropy and amplitude of magnetization is consistent with our experimental results. Our investigation of flexible BTO/LSMO bilayer ME membranes shed light on controlling the order parameters in TMOs.

## 2 Results and Discussion

Both bilayers of LSMO/BTO and BTO/LSMO bilayers were fabricated on top of sacrificial SAO‐buffered SrTiO_3_ (STO) substrates. To fabricate the freestanding membrane of the bilayer, it is essential to consider a sequence of epitaxially grown layers to determine the quality of the thin films. SAO has a cubic structure with lattice parameter *a* = 15.844 Å,^[^ ^7^ ^]^ which is approximately four times the lattice parameter of STO (*a*_STO_ = 3.905 Å). The optimized stack sequence was LSMO/BTO/SAO on STO substrates. First, we have confirmed that such a sequence provided a much higher film quality than the BTO/LSMO/SAO//STO stack, as shown by high‐resolution X‐ray diffraction (HRXRD) in Figure S1 (Supporting Information). This could be due to the relatively large lattice mismatch between BTO (*a*_BTO_ = 3.995 Å) and LSMO (*a*_LSMO_ = 3.874 Å, *f* = 3.01%) compared with that between SAO and BTO (*f* = −0.85%). The gradual increase in the lattice constant in the BTO (3.995 Å)/SAO (3.961 Å)/STO (3.905 Å) stack is critical for the fabrication of high‐quality BTO films. Next, we employed polyimide tape or a Si substrate as a support to prevent the bilayer film from dissolution (see Experimental Section for details). This process exposes the BTO layer to the air, while the LSMO is placed between the BTO and polyimide tape, serving as a bottom electrode for ferroelectric measurements.

**Figure 1:**
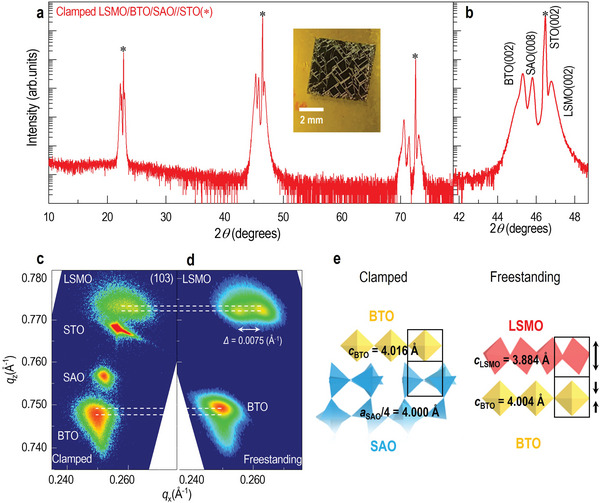
Structural characterization of heterogeneous oxide membrane. a,b) XRD 2*θ −* *ω* pattern of LSMO/BTO/SAO heterostructure on STO substrate. Inset in a) is a picture of a freestanding membrane transferred onto a polyimide tape. c,d) Reciprocal space mappings around (103)‐diffraction of clamped and freestanding layers. The membrane is attached to a SiO_2_/Si substrate for XRD alignment. The dashed lines indicate the peak shift of BTO and LSMO after the substrate is removed. e) Schematics showing the lattice deformation of layers in clamped and freestanding circumstances.

**Figure 2:**
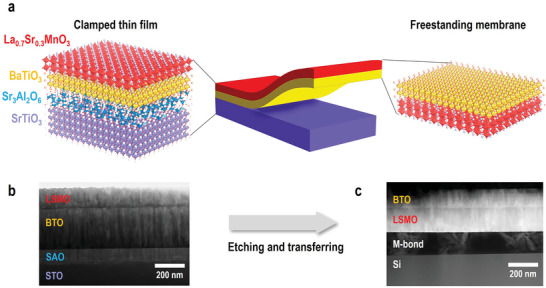
Synthesis of freestanding heterogeneous oxide membrane. a) Schematics of LSMO/BTO/SAO heterostructure on (001)‐oriented STO substrate heterostructure and freestanding BTO/LSMO membrane after dissolving SAO layer. b) A typical cross‐sectional TEM image of trilayer films on STO substrate. c) A typical STEM image of membranes glued to a silicon substrate with an M‐bond.

**Figure** **1**a,b shows the HRXRD 2*θ − ω* scans of the LSMO/BTO/SAO trilayer on (001)‐oriented STO substrates. Owing to the smaller lattice mismatching between the layers, all of the layers exhibit high‐quality epitaxial growth. The LSMO and BTO films have *c*‐axis lattice parameters of 3.879 and 3.976 Å, respectively, comparable to the lattice parameters of stoichiometric LSMO and BTO thin films on STO substrates. Note that the tetragonal phase of BTO can provide two orientations to BTO thin films, depending on the alignment of the structure, for example, *a*‐axis and *c*‐axis. XRD results reveal the coexistence of major *a*‐ and minor *c*‐ axis orientations in the pristine BTO thin film.^[^ ^8^, ^41^ ^]^ The LSMO film possesses a *c*‐axial lattice parameter close to bulk LSMO. The samples were soaked in deionized water, and the LSMO/BTO membranes were successfully separated from the substrate (see Experimental Section for details). The inset in Figure 1a shows a picture of the membrane with crack lines aligned along the diagonal directions of the STO substrates, which can be attributed to the deformation of the lattice after removing the substrate. To obtain the accurate peak shift and domain structure change before and after etching the SAO, we have performed reciprocal space mapping (RSM) around the (103) reflection for the heterostructures of LSMO/BTO/SAO on STO (Figure 1c) and the BTO/LSMO membrane (Figure 1d). The membrane was glued to a silicon substrate by M‐bond, and the RSM result of the membrane reveals that the membrane preserves the quality of each layer. The BTO in the original trilayer sample shows an elongated (103) peak, which could be due to a distribution of oxygen vacancies along the direction of the film thickness, as a mechanism to accommodate gradual strain relaxation. The *c*‐axis lattice parameter of BTO, *c*_BTO_, decreases from 4.016 Å in the trilayer to 4.005 Å in the membrane, while the in‐plane lattice parameter of BTO, *a*_BTO_, is ≈4.000 and 4.001 Å for the trilayer and membrane, respectively (Figure 1e). It is interesting to note that when BTO is released from clamping, its volume reduces. This volume reduction could come from that the amount of oxygen vacancies reduce in the BTO membrane as it is often reported that oxygen vacancy increases unit cell volume in perovskite films.^[^ ^42^ ^]^ Such a change could also be related to the strain and/or domain structure change in LSMO, as discussed below. The BTO (103) diffraction pattern in the RSM becomes a round shape for the membranes, indicating uniform stoichiometric distribution. It is noted that the center of the diffraction pattern can be assigned to *a*‐domains, and the tail can be assigned to *c*‐domains. Therefore, in the freestanding membrane, the BTO domain structure changes. The *c*‐axial lattice parameter of LSMO, *c*_LSMO_, is elongated from 3.879 to 3.884 Å (Figure 1e). LSMO also undergoes a structure change, evidenced by the obvious peak split after removing the substrate. A twin‐domain structure in LSMO appears with *Q*_x_ directional splitting of 0.0075 Å^−1^ between two peaks in the membrane. Previous reports showed twin‐domain formation in LSMO as a shear‐strain relaxation mechanism.^[^ ^43^, ^44^ ^]^ Such a twin structure has been directly observed in a plane‐view microstructure characterization.^[^ ^44^ ^]^ After removing the substrate, the crystallinity and structural quality of the LSMO twin domains increased, as evidenced by the sharp diffraction peaks. Therefore, the film quality of the BTO layer and the twin structure of the LSMO layer were improved in the membrane compared with those clamped on the substrate. Microstructural characterization was performed for both samples. **Figure** **2**a shows the illustrations of trilayer samples and membranes with atomic structures. To investigate the microstructure and interface quality, transmission electron microscopy (TEM) and scanning transmission electron microscopy (STEM) images were collected for both original trilayers and membranes. A cross‐sectional TEM image of LSMO/BTO/SAO trilayer on STO substrate is shown in Figure 2b. Dense layers with distinct interfaces are visible in the trilayer film. Figure 2c shows a cross‐sectional TEM image of the LSMO/BTO membrane glued to a silicon substrate. This bilayer membrane shows a well‐defined and flat film surface with respect to the silicon substrate, confirming the high‐quality transfer process. We have also tried to grow CoFe_2_O_4_ (CFO) films as the ferromagnetic layer on SAO as it has a higher Curie temperature. However, the surface of CoFe_2_O_4_ tends to form pyramid structures, which results in an uneven interface between CFO and BTO (See Figure S2, Supporting Information).

**Figure 3:**
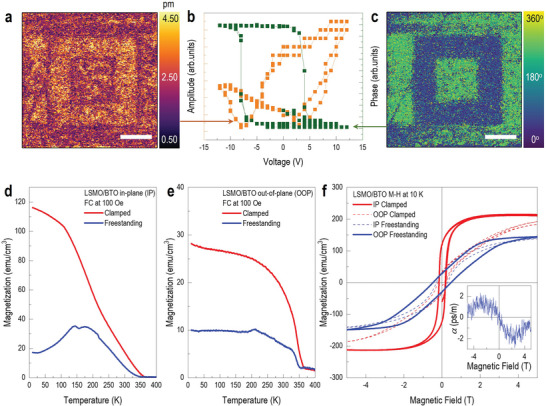
Room‐temperature ferroelectric ferromagnetism of heterogeneous membranes. a–c) PFM measurements; b) Hysteresis loops of amplitude (orange line) and phase (green line) were obtained by PFM. Box‐patterned PFM images for a) amplitude and c) phase. d)The in‐plane, and e) out‐of‐plane *M*–*T* curves for LSMO/BTO/SAO heterostructures on STO substrates (red lines) and BTO/LSMO membranes (blue lines) measured with field‐cooling at 100 Oe. f) The *M‐*‐*H* measurements were performed at 10 K. In clamped LSMO/BTO/SAO film on STO (red lines), the solid line indicates an in‐plane easy axis. Meanwhile, in the freestanding BTO/LSMO membrane (blue lines), the solid line indicates an out‐of‐plane easy axis resulting from the magnetic anisotropy reorientation. The inset shows the magnetoelectric coupling coefficient as a function of the applied DC magnetic field at 300 K.

The room‐temperature multiferroic properties of such freestanding bilayer membranes were confirmed by ferroelectric and magnetic measurements. **Figure** **3** shows the piezoresponse force microscopy (PFM) measurements of the BTO/LSMO membrane on polyimide tape. The LSMO layer serves as the bottom electrode. By applying a voltage of ±12 V, we measured the hysteresis loops and piezoresponse, as shown in Figure 3b, indicating that the room‐temperature ferroelectricity in the BTO layer is robust. We also switched the polarization of the BTO membrane layer along with the box‐patterned shape (Figure 3a,c), which further supports the realization of room‐temperature ferroelectricity.

Interestingly, the structural modulation of the LSMO is reflected in its magnetic properties. In the *M–* *T* measurement of the LSMO/BTO/SAO heterostructure on STO, the typical ferromagnetic behavior of LSMO with *T*_C_ ≈350 K was observed (red lines in Figure 3d,e).^[^ ^45^ ^]^ Note that the dimensionality and resultant shape anisotropy of the thin film spontaneously cause an in‐plane easy axis. Room‐temperature ferromagnetism still survives in the freestanding membrane despite the reduced magnetization (blue lines in Figure 3d,e). It should be remarked that a kink arises around 180–200 K in the out‐of‐plane *M*–*T* curve regardless of the thickness of LSMO, which can be attributed to the structural change of BaTiO_3_ from orthorhombic to rhombohedral at 183 K. Such a kink is an indication of the magnetoelectric coupling, as reported in other nanocomposites.^[^ ^65^ ^]^ As shown in Figure S3a (Supporting Information), the thinner LSMO comes to the stronger signal of kink, which can be interpreted in terms of the enhancement of the strain effect from the BTO layer.^[^ ^46^ ^]^ The intriguing observation is that the magnetic anisotropy reorientates from in‐plane to out‐of‐plane direction. In Figure 3f, *M*–*H* hysteresis loops exhibit anisotropy conversion. The magnetic hysteresis of the clamped film shows an in‐plane easy axis along with much higher remnant magnetization in the in‐plane orientation (solid red line), which has been reported in LSMO thin films owing to the surface anisotropy. Meanwhile, the freestanding membrane exhibits perpendicular magnetic anisotropy with larger remnant magnetization and coercive field *H*_C_ (solid blue line). We speculate that the conversion of magnetic anisotropy is attributed to the lattice elongation along the *c*‐axis observed in the XRD 2*θ − ω* scan result corresponding to the compressive strain on the LSMO (Figure 1d). Plus, owing to the heteroepitaxial arrangement between the layers, the magnetostriction of LSMO provides finite magnetoelectric coupling constant *α* at room‐temperature (inset of Figure 3f). When magnetoelectric coupling exists in materials, an oscillating magnetic field (*δH*_ac_) produces modulation of electric polarization (*δP*_ac_), and the magnetoelectric coupling constant *α* is defined as *α* = *δP*_ac_/*δH*_ac_ = *δQ*_ac_/(*δH*_ac_ × *A*), where *δQ*_ac_ and *A* are the amount of charge modulation and the area of the top electrode, respectively. The measurements were done by using a Pt top electrode and an LSMO bottom electrode (see Figure S4, Supporting Information).

The effect of lattice strain on magnetic anisotropy in LSMO thin films has been comprehensively studied.^[^ ^47^, ^48^, ^49^ ^]^ The use of various substrates with a range of lattice mismatches and orientations to modify the biaxial in‐plane strain on the thin film was an effective way to control the magnetic easy‐axis.^[^ ^49^, ^50^ ^]^ It has also been demonstrated that the microscopic strain effect induced by the step terrace structure on the surface of prepared STO substrates determined the direction of the easy axis.^[^ ^51^, ^52^, ^53^ ^]^ Interestingly, the unusual magnetic hysteresis loop shape in LSMO thin films under compressive strain has been reported, in which the direction of the spin configuration reoriented perpendicularly, similar to our magnetic results for the freestanding membrane (Figure 3).^[^ ^54^, ^55^ ^]^ The easy‐axis reorientation within the magnetic thin film is significant from the viewpoint of fundamental physics and applications for thermally stable and ultra‐high‐density devices.^[^ ^56^ ^]^ The easy‐axis reorientation of ferromagnetism was reported in LSMO:MgO nanocomposites. With increasing MgO nanoscaffold density, the *c*‐axial lattice parameter of LSMO increases, and the easy axis switches from in‐plane to out‐of‐plane.^[^ ^56^, ^57^ ^]^ It should be noted that all previous reorientations of magnetic anisotropy require external strain from the substrate or touching material. Hence, the freestanding membrane offers another route to the spontaneous emergence of the anisotropy reorientation. The reorientation of magnetic anisotropy consistently occurs even in the other freestanding membranes with different thicknesses of LSMO (Figure S3, Supporting Information).

**Figure 4:**
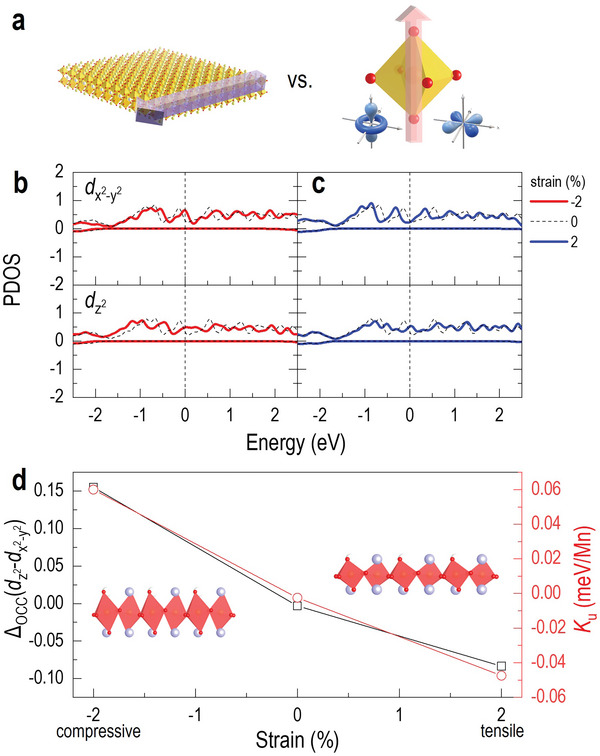
Magnetic anisotropy reorientation induced by an orbital occupancy modulation. a) Schematic of competition between the two sources for magnetic anisotropy, shape anisotropy, and strain anisotropy. b,c) The calculated projected density of states of $d_{x^{2}-y^{2}}$ and $d_{z^{2}}$ orbitals for compressive strain (− 2%) case (solid red line) and tensile strain (2%) case (solid blue line). The dashed black lines indicate an unstrained case. d) By using the orbital occupancies, we further calculate the orbital occupancy difference between $d_{x^{2}-y^{2}}$ and $d_{z^{2}}$ orbitals (black) and magnetic anisotropy induced by a strain effect (red) with respect to the applied strain.

We attempted to interpret the magnetic anisotropy conversion induced by strain modulation in terms of the orbital occupancy via first‐principles spin‐polarized density functional theory calculation.^[^ ^58^ ^]^ The strain anisotropy helps the LSMO thin film to have distinct magnetic properties beyond the effect of surface anisotropy from two‐dimension nature (**Figure** **4**a). Figure 4b,c show the calculated spin‐resolved projected density of states (PDOS) for specific orbitals of Mn atom, which supports that the magnetic properties of LSMO mainly originate from the $d_{x^{2}-y^{2}}$ and $d_{z^{2}}$ orbitals because the other *d*_xy_, *d*_yz_ and *d*_zx_ orbitals around Fermi energy (*E*_F_) are empty (Figure S5, Supporting Information). In particular, the $d_{x^{2}-y^{2}}$ and $d_{z^{2}}$ orbitals are influenced by biaxial strain, −2% (compressive, solid red line), 0% (unstrained, dashed line), and 2% (tensile, solid blue line). We suppose that LSMO under compressive strain has *a*_LSMO_ = 3.79 Å and *c*_LSMO_ = 3.93 Å, and vice versa under tensile strain. In unstrained LSMO, the PDOS for $d_{x^{2}-y^{2}}$ and $d_{z^{2}}$ orbitals are almost identical from −2.5 to 2.5 eV, which does not generate any magnetic anisotropy. On the other hand, tensile and compressive strains change the orbital occupancies of $d_{x^{2}-y^{2}}$ and $d_{z^{2}}$ in the opposite manner. The tensile strain dramatically enhances the relative occupancy of $d_{x^{2}-y^{2}}$ around *E*_F_, but weakens that of $d_{z^{2}}$. The calculated magnetic anisotropy energy (*K*_u_) shows a similar trend to the orbital occupancy difference between $d_{x^{2}-y^{2}}$ and $d_{z^{2}}$, *Δ*_OCC_($d_{z^{2}}\;-\;d_{x^{2}-y^{2}}$), as shown in Figure 4d, and the LSMO under tensile strain (2%) gives a negative *K*_u_ (see Experimental Methods for details). This effectively corresponds to the magnetic properties of a typical LSMO film with surface anisotropy. Meanwhile, *K*_u_ linearly increases with decreasing compressive strain (−2%), which indicates the possible conversion of magnetic anisotropy into an out‐of‐plane easy axis. The observed elongation of the *c*‐axial lattice parameter of the LSMO layer by 0.3% (Figure 2b) generates an effect akin to strain anisotropy and leads to anisotropy reorientation, as illustrated in Figure 3c. Since the absolute value of *K*_u_ is highly associated with the shape of the *M*–*H* hysteresis loop as *K*_u_ ∝ *H*_c_ *M*_s_, an increase in anisotropy can give rise to a sizeable coercive field *H*_c_, consistent with our magnetic characterization experiments.

## 3 Conclusion

This study reports the realization of a freestanding oxide bilayer membrane with room‐temperature ferroelectricity and ferromagnetism. Compared with the substrate‐clamped films, the quality of the BTO layer in the membrane was improved by removing the strain effect. We confirmed the ferroelectricity and ferromagnetism in the BTO/LSMO bilayer membrane, which implies that the fabrication of a freestanding membrane by dissolving the water‐soluble SAO layer does not destroy the original long‐range ordering of the materials. Both the ferroelectric and magnetic domains were strongly modulated by removing substrate strain. This work opens up new avenues for the design of multiferroic freestanding oxide membranes for ME effect and spintronic applications.

## 4 Experimental Section

### Thin‐Film Growth

Epitaxial Sr_3_Al_2_O_6_, BaTiO_3_, and La_0.7_Sr_0.3_MnO_3_ thin films were grown on atomically flat (001)‐oriented single‐crystalline SrTiO_3_ substrates using pulsed laser epitaxy (PLE, KrF excimer laser, *λ* = 248 nm) at 700 °C. Sr_3_Al_2_O_6_ thin films were grown under 100 mTorr, and BaTiO_3_ and La_0.7_Sr_0.3_MnO_3_ thin films under 50 mTorr of oxygen . Laser fluence of 2, 1.2, and 1.5 J cm^−2^ were also applied for Sr_3_Al_2_O_6_, BaTiO_3_, and La_0.7_Sr_0.3_MnO_3_, respectively. After growth, the films were cooled down to room temperature at 200 Torr of oxygen.

### Freestanding Membrane Fabrication

After deposition, the samples were cut ≈1 × 1 mm^2^ in size and transferred onto flexible Kapton tape (polyimide, PI) and a glass slide. After 48 h of soaking submerged in DI water, the exfoliation process was complete, and the flexible freestanding LSMO/BaTiO_3_/PI membranes could be extracted. The PI substrate has a 25 µm thickness. For transport measurements and microstrcture characterization, the membranes have also been transferred on top of silicon substrates (Figure 1b). To avoid dissolution of the membranes, M‐bond was employed and the result of the transmission electron microscope showed it fastens the LSMO/BaTiO_3_ layers nicely.

### Film Properties Characterization

The structure of these heterostructures was investigated by x‐ray diffraction (XRD, Panalytical MRD PRO X‐ray diffractometer). The reciprocal space maps (RSM) for the freestanding membranes were done by another XRD (Rigaku Smartlab). The magnetism was measured using a Vibrating Sample Magnetometer (Quantum Design). The in‐plane magnetization versus field (*M*–*H*) loops were measured at 10 K with the magnetic field applied both parallel and perpendicular to the film plane. The magnetization versus temperature (*M*–*T*) curves were measured during heating from 10 to 400 K after a field cooling from above the *T*_C_ down to 10 K. A magnetic field of 100 Oe was used for the cooling and measuring field. The magnetoelectric coupling coefficient *α* was measured using a homemade magnetoelectric susceptometer and the physical property measurement system (Quantum Design) to apply a DC magnetic field.^[^ ^28^ ^]^ The AC magnetic field was applied by a pair of counter‐wound solenoid coils, which compensated for the stray magnetic field outside the solenoid coils and minimized the magnetic field‐induced electrical noise. The *δH*_ac_ of 1 Oe oscillated at a frequency of 237 Hz.

### Piezoelectric Response Characterization

All PFM measurements were captured using Pt‐coated cantilevers with a spring constant of ≈2 N m^−1^ and a resonance frequency of ≈75 kHz unless otherwise stated. The PFM measurements were performed on a commercial Cypher AFM (Asylum Research, Santa Barbara, CA) with an integrated interferometer in the form of a laser Doppler vibrometer system (Polytec GmbH, Waldbronn, Germany) to achieve highly sensitive electromechanical imaging and spectroscopy by interferometric displacement sensing (IDS)‐PFM. IDS‐PFM was captured using 1Vac at 30 kHz. Box patterned poling was achieved by the application of (+/‐) 15 V DC to the conductive probe in contact with the surface. PFM hysteresis loops were captured using switching spectroscopy Band excitation (BE)‐ PFM achieved by coupling the AFM with an external arbitrary wave generator and data acquisition electronics based on a NI‐6115 fast DAQ card. Custom software was used to generate the probing signal and store local BE and hysteresis loops. All measurements were carried out at room temperature and at 30–40% relative humidity.

### Characterization of Microstructure

TEM specimens were prepared using focused ion beam (FIB) milling (30 kV, 30 nA Ga beam) with a lift‐out procedure, which was carried out in an FEI Helios 600 FIB/SEM system. TEM characterization was conducted on an FEI Tecnai F30 microscope with an acceleration voltage of 200 kV.

### Orbital Occupancy Calculation (DFT)

The generalized‐gradient approximation‐Perdew–Burke–Ernzerhof‐sol (GGA‐PBEsol) exchange‐correlation functionals^[^ ^59^ ^]^ and projector‐augmented wave method^[^ ^60^ ^]^ were used with plane wave basis, as implemented in the Vienna Ab Initio Package code.^[^ ^61^, ^62^ ^]^ For the electronic structure of La_0.75_Sr_0.25_MnO_3_, a Hubbard *U* correction was used with *U* = 4 eV to the strongly localized Mn‐3*d* state. The plane waves were included up to the kinetic‐energy cutoff of 520 eV. For the Brillouin‐zone integration, Γ‐centered 8 × 8 × 6 *k*‐point meshes were used. The calculations were converged in energy to 10–6 eV and atomic positions were fully relaxed until the residual forces on each atom was less than 0.001 eV Å^−1^. To calculate the magnetic anisotropy energy (*K*_u_), the spin‐orbit coupling (SOC) was included in a noncollinear mode,^[^ ^63^, ^64^ ^]^ using an energy convergence threshold of 10–8 eV.

The *K*_u_ is defined as

(1) $$K_{u}=E_{x}-E_{z}=\sum_{u,o,\sigma ,\sigma^{\prime }}{(-1)}^{1\;-{\;\delta }_{\sigma \sigma^{\prime }}}\left[\frac{{\left|o^{\sigma^{\prime }}\left|L_{z}\right|u^{\sigma }\right|}^{2}\;-\;{\left|o^{\sigma^{\prime }}\left|L_{x}\right|u^{\sigma }\right|}^{2}}{E_{u}^{\sigma }\;\;-{\;\;E}_{o}^{\sigma^{\prime }}}\right]$$

where *u^*σ*^* and *o^*σ*^* ^′^ indicate the unoccupied and occupied eigenstate, respectively (*σ* and *σ*′ represent the spin state). $E_{u}^{\sigma }$ and $\;E_{o}^{\sigma^{\prime }}$ denote the eigenvalue of unoccupied and occupied d‐orbital states. “*ξ*” and *δ* are the SOC coefficients and the Kronecker delta function. The *K*_u_ was obtained from the difference between the total energy of calculated results with *E*_x_ and *E*_z_. (here *E*_x_ and *E*_z_ indicate the total energy with the spin direction along the (100) axis and (001) axis, respectively.

## Conflict of Interest

The authors declare no conflict of interest.

## Supporting information

Supporting Information

## Data Availability

The data that support the findings of this study are available from the corresponding author upon reasonable request.
