# Supplementary material for: Heterogeneous Integration of Freestanding Bilayer Oxide Membrane for Multiferroicity
Source: Adv Sci (Weinh). 2023 Apr 3;10(15):2207481. doi: 10.1002/advs.202207481 (PMC10214221; doi:10.1002/advs.202207481)
Supplement: Supplementary file 1 — Supporting Information [file ADVS-10-2207481-s001.pdf]

ADVANCED SCIENCE
Open Access

Supporting Information

for *Adv. Sci.*, DOI 10.1002/advs.202207481

Heterogeneous Integration of Freestanding Bilayer Oxide Membrane for Multiferroicity

Kyeong Tae Kang, Zachary J Corey, Jaejin Hwang, Yogesh Sharma, Binod Paudel, Pinku Roy, Liam Collins, Xueijing Wang, Joon Woo Lee, Yoon Seok Oh, Yeonhoo Kim, Jinkyoung Yoo, Jaekwang Lee, Han Htoon, Quanxi Jia and Aiping Chen**

Supporting Information

Heterogeneous integration of freestanding bilayer oxide membrane for multiferroicity

Kyeong Tae Kang, Zachary J Corey, Jaejin Hwang, Yogesh Sharma, Binod Paudel, Pinku Roy, Liam Collins, Xueijing Wang, Joon Woo Lee, Yoon Seok Oh, Yeonhoo Kim, Jinkyoung Yoo, Jaekwang Lee, Han Htoon, Quanxi Jia, and Aiping Chen**

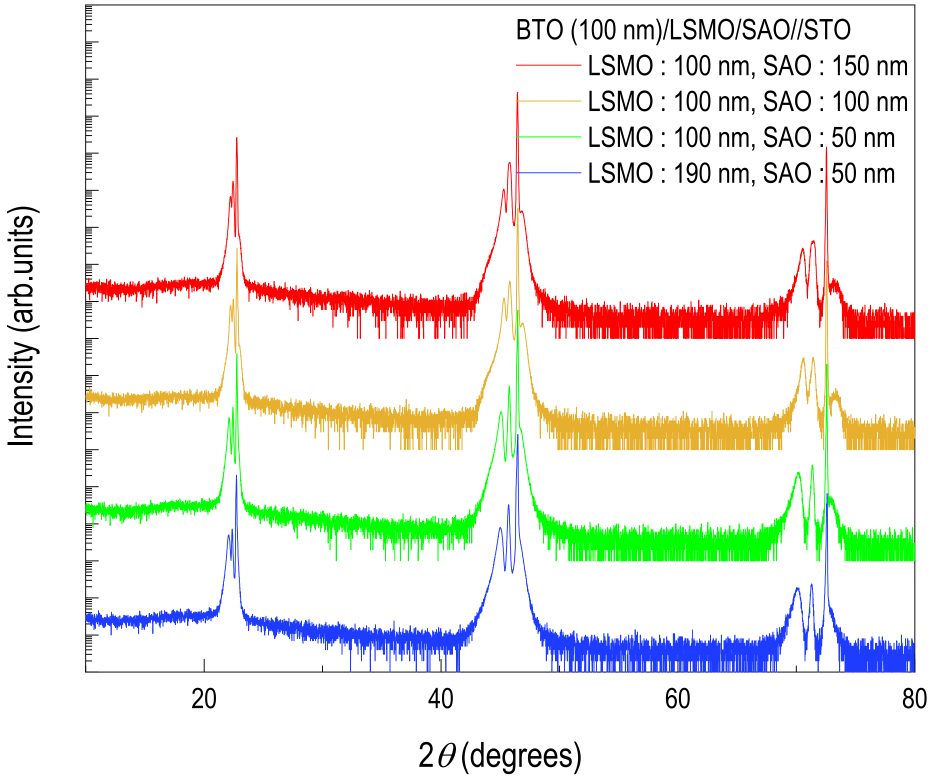

Figure S1. X-ray diffraction 2θ-ω pattern of BTO/LSMO/SAO heterostructure on STO substrate with various thicknesses of layers.

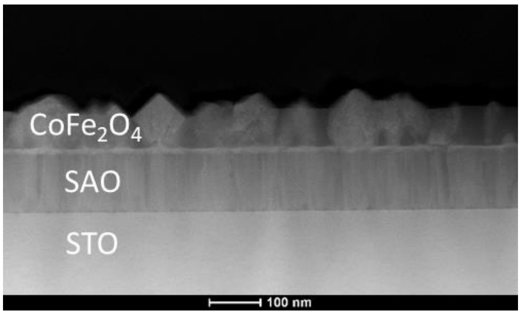

Figure S2. Cross-sectional image of a typical $CoFe_2O_4/Sr_3Al_2O_6$ heterostructure on $SrTiO_3$ substrate.

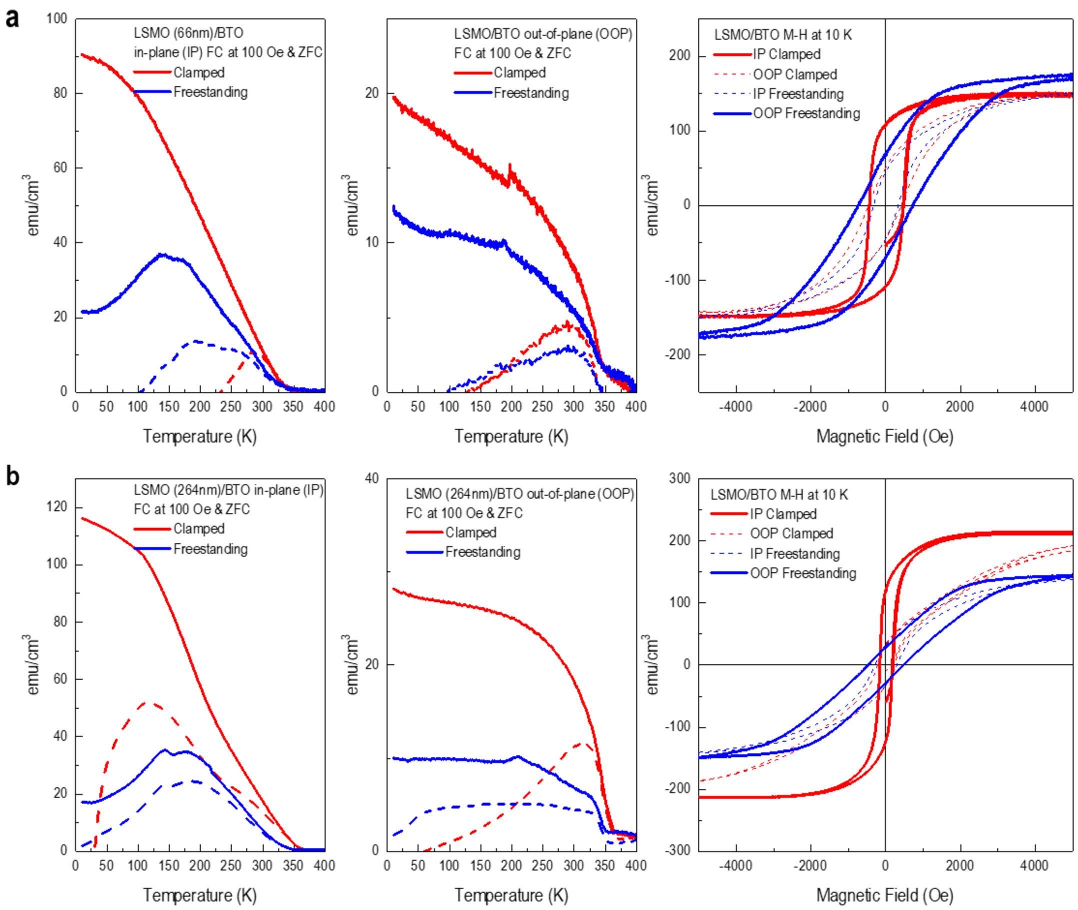

Figure S3. The in-plane and out-of-plane *M-T* curves and the *M-H* hysteretic loops for LSMO/BTO/SAO heterostructure on STO substrate (red lines) and BTO/LSMO membrane (blue lines) with different thicknesses of LSMO. The heterostructure of a) has LSMO of 66 nm and of b) does LSMO of 264 nm).

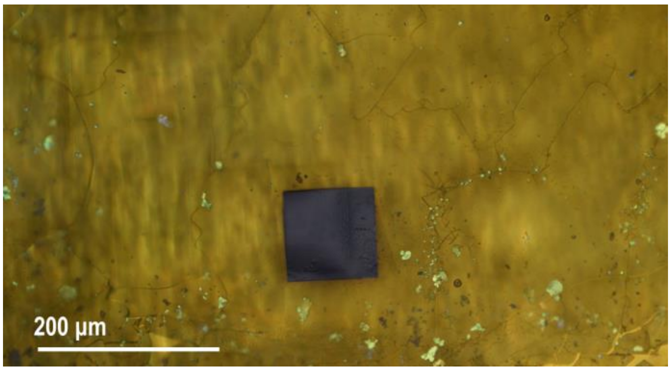

Figure S4. Image of Pt electrode of 100 µm length deposited on BTO/LSMO freestanding membrane. We have performed a magnetoelectric coupling coefficient measurement as depicted in the inset of Figure 3f. To avoid the cracks generated on the membrane surface, we deposited Pt electrodes of 100 µm length. The magnetoelectric coupling coefficient α was measured using a homemade magnetoelectric susceptometer and the physical property measurement system (Quantum Design) to apply DC magnetic field. The δH_{ac} of 1 Oe oscillates at a frequency of 237 Hz.

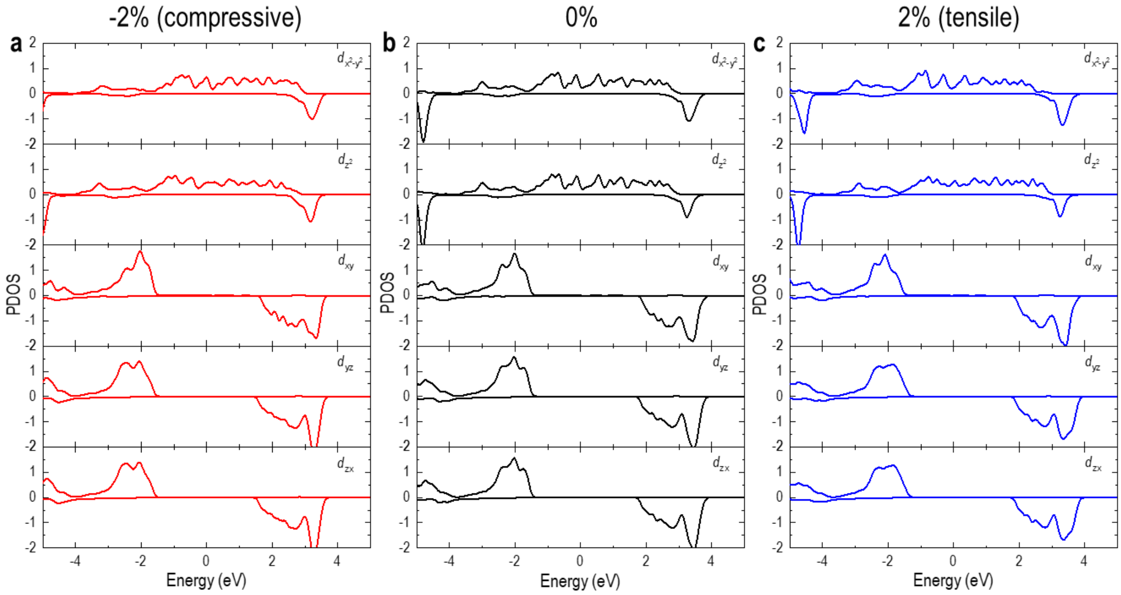

Figure S5. The calculated projected density of states showing orbital occupancies of Mn atom. a) -2% compressive, b) 0% unstrained, and c) 2% tensile strain cases each.
